# Ecological Momentary Assessment of Voice & Psychological Factors: Group & Individual Mechanisms

**DOI:** 10.1002/lary.70049

**Published:** 2025-08-15

**Authors:** Stephanie Misono, Viann N. Nguyen‐Feng, Xiaofan Lei, Erin Feddema, Anika Tella, Ali Stockness, Patricia A. Frazier, Erich Kummerfeld, Kelvin O. Lim

**Affiliations:** ^1^ Department of Otolaryngology University of Minnesota Twin Cities Minneapolis Minnesota USA; ^2^ Department of Psychology University of Minnesota Duluth Duluth MN USA; ^3^ Magnit Maple Grove MN USA; ^4^ Boston Scientific Minneapolis MN USA; ^5^ Department of Otolaryngology University of Minnesota Twin Cities Minneapolis MN USA; ^6^ Department of Psychology University of Minnesota Twin Cities Minneapolis MN USA; ^7^ Institute for Health Informatics University of Minnesota Twin Cities Minneapolis MN USA; ^8^ Department of Psychiatry and Behavioral Sciences University of Minnesota Twin Cities Minneapolis MN USA

**Keywords:** causal modeling, ecological momentary assessment, psychology, voice

## Abstract

**Objectives:**

Cross‐sectional associations between voice and psychological factors are known, but changes over time offer opportunities to refine our understanding of their interactions and consider customized treatment options. Study objectives were to measure relationships between voice and psychological factors using ecological momentary assessment and applying (1) group‐level time series analysis and (2) group and (3) individual causal modeling to identify key psychological factors relevant for voice outcomes.

**Methods:**

Adults (*N* = 32) with primary muscle tension dysphonia completed multiple assessments daily for 10 days. Measures included items from the Voice Handicap Index‐10, voice‐adapted perceived present control scale, items from NIH PROMIS and the NIH Toolkit to assess distress, and the Positive and Negative Affect Scale. Group‐level time series analysis was conducted using dynamic structural equation modeling; causal analysis utilized the Greedy Fast Causal Inference algorithm.

**Results:**

In group‐level time series analyses, neither perceived control nor distress predicted subsequent timepoint voice handicap scores. In group‐level causal modeling, anxiety was causal for voice handicap, but perceived control was not. Individual‐level analyses identified various causal factors for voice handicap including perceived control and negative affect, and to a lesser extent, serenity, anxiety, somatic arousal, and stress.

**Conclusions:**

Group‐level analyses may obscure important heterogeneity that is identifiable using individual‐level causal analyses. For example, perceived control was not identified as predictive or causal for voice handicap at the group level; but was a salient causal factor for voice handicap in some individuals. Causal modeling using intensive longitudinal datasets offers a potential avenue for individualized treatment approaches.

**Level of Evidence:**

4.

## Graphical abstract

Adults (*N* = 32) with primary muscle tension dysphonia completed multiple assessments daily for 10 days. In group‐level causal modeling, anxiety was causal for voice handicap, but perceived control was not. Individual‐level analyses identified various causal factors for voice handicap including perceived control and negative affect, and to a lesser extent, serenity, anxiety, somatic arousal, and stress.

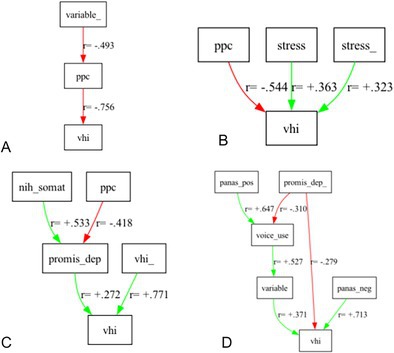

## 1 Introduction

A growing body of literature examines the intersection of voice and psychology. Distress (e.g., depression, anxiety, stress) is common among patients with voice disorders, with distress severity comparable to that of outpatients at cancer clinics [1, 2]. Further, mental health concerns affect care‐seeking and care outcomes in people with voice disorders [3]. One focus area has been the relationship between voice handicap (measured by the Voice Handicap Index‐10 (VHI‐10) [4]) and voice‐related present perceived control (PPC) (measured by the voice‐adapted version of the present subscale of the perceived control scale) [5, 6]. Perceived control is the extent to which a person feels they can control a problem or their response to the problem. A moderate negative correlation between these two factors has been observed in multiple studies [5, 7, 8], but cross‐sectional associations cannot shed light on temporal patterns or causality. Therefore, interest is growing in examining both voice and psychological measures over time. A small longitudinal study, albeit with only two timepoints, indicated that perceived control was more predictive of subsequent voice handicap than vice versa [8].

Ecological momentary assessment (EMA) is a repeated data collection methodology in which participants provide real‐time data in their typical environments, reducing recall bias and increasing generalizability [9]. By identifying temporal change patterns, EMA can suggest variables to target in interventions. For example, if changes in variable A precede changes in the outcome of interest, and changes in the outcome of interest precede changes in variable B, then variable A would be a more logical target for intervention. EMA has begun to be applied in voice research to identify temporal patterns of potential interest between key variables. For example, in a prior small study, EMA was used to repeatedly measure voice handicap and perceived control [10]. However, the study was limited in collected timepoints (i.e., 14) and number of participants (*n* = 15), and a universal temporal pattern was not identified.

Traditional analysis of EMA data has been at the group level and has been correlative [10]. Newer analytic approaches now allow *causal* analysis using repeated assessments. By combining graph theory, statistics, and machine learning to produce hypothetical causal models [11], these analyses go beyond identifying temporal patterns between variables of interest to determining which variables are causal for other variables [12, 13]. Increased analytic capacity made possible by machine learning allows consideration of all possible models for a set of measures and determination of which causal model is most justified by the data. In a further innovation, with sufficient data, these analyses can be applied at the individual level, allowing the identification of important causal pathways within individuals and raising the possibility of personalized intervention [14, 15].

The current study built on prior EMA work by increasing both participants and collected timepoints to facilitate more robust modeling. Additional relevant measures were included, including distress and variables related to voice use/context. Finally, the study applied causal analytic techniques to identify causal models linking variables of interest at the individual level.

With prior literature as a foundation [8], the following hypotheses were tested: (1) in time series analyses at the group level, (a) perceived control and (b) distress (e.g., anxiety, depression) would predict subsequent voice handicap; (2) group causal analysis would identify perceived control and distress as causal for voice handicap; and (3) individual causal models would demonstrate heterogeneity with regard to factors causal for voice handicap, the primary outcome.

## 2 Materials and Methods

### 2.1 Enrollment and Study Procedures

Adult patients with primary muscle tension dysphonia were identified from otolaryngology clinics in the US Midwest by their treating providers and screened for eligibility and interest by the research team. Primary muscle tension dysphonia was chosen to avoid structural abnormalities that could potentially create a floor effect on voice handicap scores. Among those who consented, 5 people did not initiate study participation, and 1 withdrew after contracting COVID‐19, leaving 32 participants. Subjects were provided iPod touch study devices and were required to have reliable internet access and be able to independently complete questionnaires in English. Participants completed a mean of 83% (SD 23%) of all possible assessments. Measures (listed below) were collected four times daily within 1 h intervals set 4 h apart (i.e., 3 times during the day and once in the evening) for 10 days of repeated measures. Response windows closed after 90 min to minimize recall bias. Responses were collected using REDCap linked to a text messaging service alerting participants when it was time to complete an assessment. Additional optional “anytime” surveys were also completed by participant initiation and included in causal data analysis. Participants were given gift cards to compensate for their time. The study was approved by the Institutional Review Board (#1703 M09221).

### 2.2 Measures

All momentary measures were prefaced with an instruction to respond reflecting experiences/feelings since the last check‐in.

#### 2.2.1 Voice Measures

Items from the VHI‐10 [4] were used to measure voice handicap (i.e., emotional, functional, and physical impacts of a voice disorder). Because using the entire scale at every timepoint risks response fatigue, the item from each domain (emotional (#9), functional (#4), physical (#6)) most closely related to VHI‐10 total scores in prior data was used [8].

Participants were also asked: (a) how much they used their voice since their last check‐in (1 = never to 5 = a great deal); (b) the amount of background noise while speaking or singing, if applicable (1 = no background noise to 4 = high level noise); and (c) the variability of voice quality since their last check‐in (1 = not at all to 5 = extremely).

#### 2.2.2 Perceived Control

Voice‐related perceived control was measured using the 8‐item voice‐adapted present control subscale of the Perceived Control over Stressful Events Scale [5, 16]. Conventions for selecting at least three items per construct for micro‐longitudinal assessment items were followed [17]. Based on prior factor analysis [8], three of four of the positively‐worded items were selected (#2, 5, and 7). The fourth was not selected because it contained a conditional emotion (“upset”).

#### 2.2.3 Distress

Depression was measured with the PROMIS Emotional Distress–Depression 4‐item short form [18], and anxiety was measured with the PROMIS Emotional Distress–Anxiety 4‐item short form [19]. Arousal was measured with the NIH Toolbox Fear–Somatic Arousal 6‐item fixed form [20]. Items were rated on a 1 (not at all) to 5 (extremely) scale. Three items from this scale (e.g., “I felt dizzy or lightheaded”) were selected for the repeated assessments [21, 22]. Participants also rated their current stress level (0 = not at all to 5 = extreme) [23].

Positive and negative affect were measured with the 10‐item International Positive and Negative Affect Schedule Short Form on a 1 (never) to 5 (always) scale [24]. Three positive (“determined,” “attentive,” “alert”) and three negative (“upset,” “nervous,” “afraid”) subscale items were selected for the repeated assessments based on prior research demonstrating high relevance with voice and psychological outcomes [10, 25]. Serenity was measured with the three‐item PANAS‐X Serenity subscale [26]. Items (e.g., “calm”) were rated on a 1 (very slightly or not at all) to 5 (extremely) scale.

### 2.3 Group‐Level Analysis Plan

Preliminary analyses were conducted with RStudio version 2024.04.2+764 [27]. Little's Missing Completely at Random (MCAR) [28] test was used to examine missingness using the *mcar_test* function in the *naniar* R package [29]. Variabilities between EMA timepoints between and within group were calculated with coefficient omegas using the *omegaSEM* function in the *multilevelTools* R package [30]. These multilevel reliabilities were used to demonstrate how much scale items varied within scales and within persons across timepoints [31, 32]. Statistical significance was determined using 95% confidence intervals.

For Hypothesis 1, dynamic structural equation modeling (DSEM) was used to create latent factors and account for time lag [33, 34]. All constructs with composite scores were included in DSEM. Variables were parsed into latent within‐ and between‐person factors. Variables were also lagged by 1 timepoint to control for and estimate the effect of a prior observation on the next observation, to address Hypothesis 1. Analyses were conducted in Mplus version 8 using a Bayes full‐information estimator recorded from each tenth of 1000 Markov Chain Monte Carlo iterations [35]. Level 1 was the within‐person effect of time, and Level 2 was the between‐person effect allowing for correlated error terms. Level 1 comprised two autoregressive paths (x_t_ on x_t‐1_; y_t_ on y_t‐1_) and two cross‐lagged paths (x_t_ on y_t‐1_; y_t_ on x_t‐1_) with random slopes on the aforementioned four paths.

Since voice handicap may increase throughout the day (i.e., higher evening than morning scores) [10], exploratory DSEM was also conducted with daytime scores averaged. These exploratory analyses examined whether daytime scores predicted evening scores. Cohen's conventions were used for effect size measures [36].

### 2.4 Causal Analysis Plan

Causal discovery analysis was conducted both at the group level and for each participant's data to address Hypotheses 2 and 3. Greedy Fast Causal Inference analysis was performed using causal‐cmd 1.8.0 from the Center for Causal Discovery [37]. Because the analysis algorithm drops participants if they have any missing data, missing values were imputed using mean imputation only if > 80% of items in both that timepoint and in the given variable were completed [38]. Less than 1% of the values were imputed (327 of 39,680 possible values) from among the completed assessments. Alpha was set at 0.05. All variables and their prior values (denoted by “_” following the variable name) were included in the analyses. The causal analyses focused on factors potentially causal for voice handicap, including perceived control and the other psychological factors examined in the group‐level time series analyses. On an exploratory basis, single item variables such as voice use, discomfort, variability, and background noise were included in the causal analyses.

## 3 Results

### 3.1 Participants and Descriptive Analyses

Thirty‐two adults (mean age 52 years, SD 15.5 years) with primary muscle tension dysphonia participated, including 87.5% cisgender women and 12.5% cisgender men. 90.6% of participants were White, 6% Black/African American, and 3.1% identified as both Multiracial and Hispanic/Latino/Spanish origin. All participants completed at least a high school degree or equivalent.

Mean baseline total VHI‐10 was 18.56 (SD 9.83). Mean baseline voice‐related perceived present control (PPC) score was 2.89 (SD 0.67). 21.9% reported prior and 75% current participation in speech/voice therapy; 25% reported current and 65.6% prior participation in psychological therapy. Mean voice problem duration was 3.60 (SD 4.60) years.

### 3.2 Group‐Level Time Series Findings

MCAR testing indicated timepoint‐to‐timepoint data were missing completely at random (*χ* ^2^ [14] = 12.79, *p* = 0.54). Table 1 lists timepoint‐to‐timepoint reliabilities. Between‐group omegas were all considered acceptable (0.75 < *ω* < 0.99). Within‐group omegas were relatively low (0.57 < *ω* < 0.89), indicating fluctuations in how items covary within a person across time and suggesting that items within a construct covered different experiences, eliciting different responses at different occasions [31]. Omegas for voice handicap, arousal, and negative affect were statistically similar to each other and significantly lower than the remaining constructs (perceived control, depression, anxiety, positive affect, serenity).

**TABLE 1:** Timepoint‐to‐timepoint reliabilities.

Construct	*ω*_between_			*ω*_within_		
	Est.	95% CI LL	95% CI UL	Est.	95% CI LL	95% CI UL
Voice handicap	0.909	0.850	0.967	0.568	0.512	0.623
Perceived control	0.967	0.947	0.988	0.748	0.721	0.774
Depression	0.956	0.930	0.982	0.742	0.716	0.769
Anxiety	0.956	0.931	0.982	0.746	0.721	0.772
Arousal	0.754	0.606	0.901	0.596	0.553	0.639
Negative affect	0.960	0.933	0.988	0.660	0.621	0.699
Positive affect	0.973	0.955	0.991	0.816	0.796	0.836
Serenity	0.991	0.985	0.997	0.891	0.879	0.902

*Note: N* = 32.

Abbreviations: CI = confidence interval, Est. = estimate, LL = lower limit, UL = upper limit.

Regarding reliabilities, group level DSEM analyses indicated that prior‐timepoint (*t*–1) scores moderately predicted subsequent‐timepoint (*t*) scores for each measure of interest (e.g., prior‐timepoint voice handicap predicted subsequent‐timepoint voice handicap, *β*_voice handicap_ = 0.267, 95% CI [0.185, 0.353]), suggesting moderate stability between timepoints across participants. Across all models, variables were statistically similar in the strength of this predictive relationship; *β*s = 0.243–0.300.

Regarding Hypothesis 1a, prior‐timepoint perceived control scores did not predict subsequent‐timepoint voice handicap scores (*β* = −0.040, 95% CI [−0.145, −0.050]). Regarding Hypothesis 1b, psychological distress variables all had small and mostly nonsignificant effect sizes. Prior‐timepoint serenity scores predicted subsequent‐timepoint voice handicap scores (*β* = −0.124, 95% CI [−0.205, −0.052]) in the expected direction. The remaining measures (i.e., anxiety, arousal, depression, affect) did not have significant timepoint‐to‐timepoint lag effects with voice handicap. Table 2 lists the *β* coefficients summarizing relationships between predictor variables and voice handicap.

**TABLE 2:** Prior timepoint prediction of subsequent timepoint voice handicap scores.

Construct	*β*		
	Est.	95% CI LL	95% CI UL
Perceived control	−0.040	−0.145	−0.050
Depression	0.044	−0.065	0.173
Anxiety	0.069	−0.028	0.167
Arousal	0.102	−0.005	0.211
Negative affect	0.030	−0.054	0.117
Positive affect	−0.041	−0.126	0.041
Serenity	−0.124	−0.205	−0.052

*Note: N* = 32.

Abbreviations: CI = confidence interval, Est. = estimate, LL = lower limit, UL = upper limit.

In exploratory analyses examining how averaged daytime scores predicted subsequent evening scores, MCAR testing indicated these data were missing at random (*χ* ^2^ [7] = 4.59, *p* = 0.71). Exploratory analyses identified modest effect sizes. The model testing daytime serenity score on evening voice handicap was replicated (*β* = −0.124, 95% CI [−0.247, −0.009]). The model testing daytime perceived control scores predicting evening voice handicap scores was significant in the expected direction (*β* = −0.184, 95% CI [−0.331, −0.030]), indicating that higher daytime voice‐related perceived control scores were associated with lower evening voice handicap scores. All other effects remained nonsignificant.

### 3.3 Group‐Level Causal Analysis Findings

**FIGURE 1:**
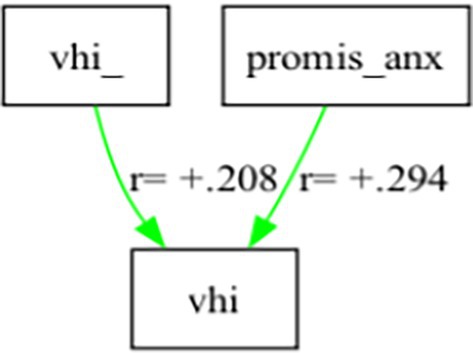
Graph showing group‐level causal modeling. Prior vhi (“vhi_”) and anxiety were causal for voice handicap (“vhi”) at the group level. [Color figure can be viewed in the online issue, which is available at www.laryngoscope.com]

At the group level, regarding Hypothesis 2, causal analysis identified anxiety and prior VHI (“vhi_”), but not perceived control, as being causal for vhi (Figure 1).

### 3.4 Individual Causal Analysis Findings

**FIGURE 2:**
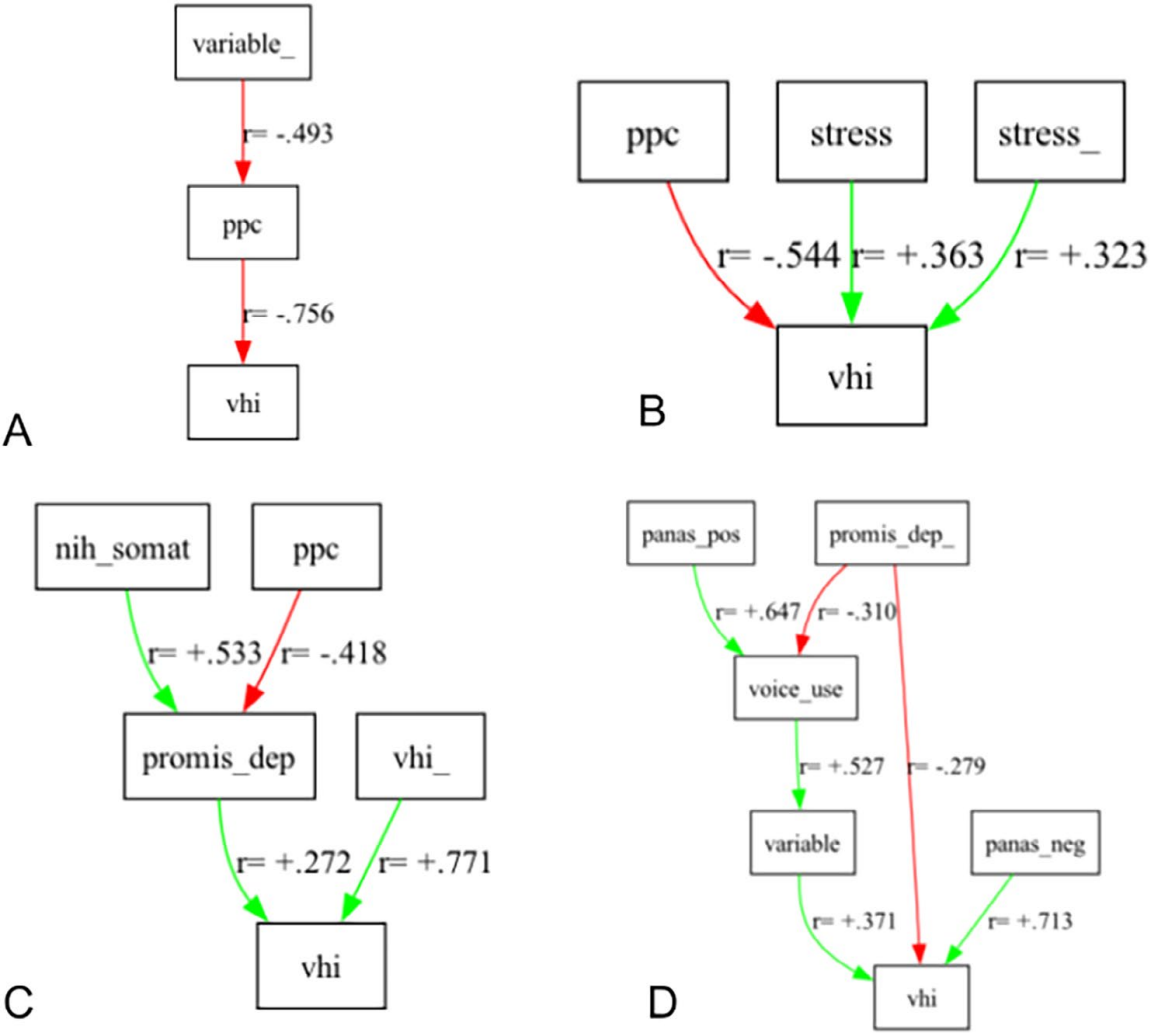
Examples of individual causal models identifying factors that are causal for voice handicap, and to what extent, as reflected by the magnitude of the r for each causal pathway. The +/− sign of the r coefficient indicates whether the causality was positive or negative (i.e., inverse). In panel (A) (subjectID 19), prior variability (“variable_”) of voice quality was causal for perceived control (“ppc”), which was in turn causal for voice handicap (“vhi”). In panel (B) (subjectID 3), perceived control, stress, and prior stress (“stress_”) were causal for voice handicap, with perceived control having the strongest effect. In panel (C) (subjectID 38), perceived control and somatic arousal were both causal for depression, which in turn was causal for voice handicap along with prior voice handicap (“vhi_”). In panel (D) (subjectID 6), a more complex model is shown, with multiple factors causal for voice handicap including prior depression (“promis_dep_”), which was causal for voice handicap both indirectly and directly; voice quality variability, and negative affect (“panas_neg”). [Color figure can be viewed in the online issue, which is available at www.laryngoscope.com]

Twenty‐eight participants had sufficient data for individual causal analysis. Analyses identified factors causal for VHI in 23 participants. The most salient psychological factors were negative affect and perceived control, and to a lesser extent, serenity, anxiety, somatic arousal, and stress. Salient voice‐related factors included discomfort, voice use, and voice variability. Prior voice handicap, anxiety, stress, discomfort, and perceived control were variably identified as important causal factors of voice handicap. For illustration, some sample individual causal models of increasing complexity are presented (Figure 2), showing examples of how different factors were causal for voice handicap, and the strengths of those causal relationships.

**FIGURE 3:**
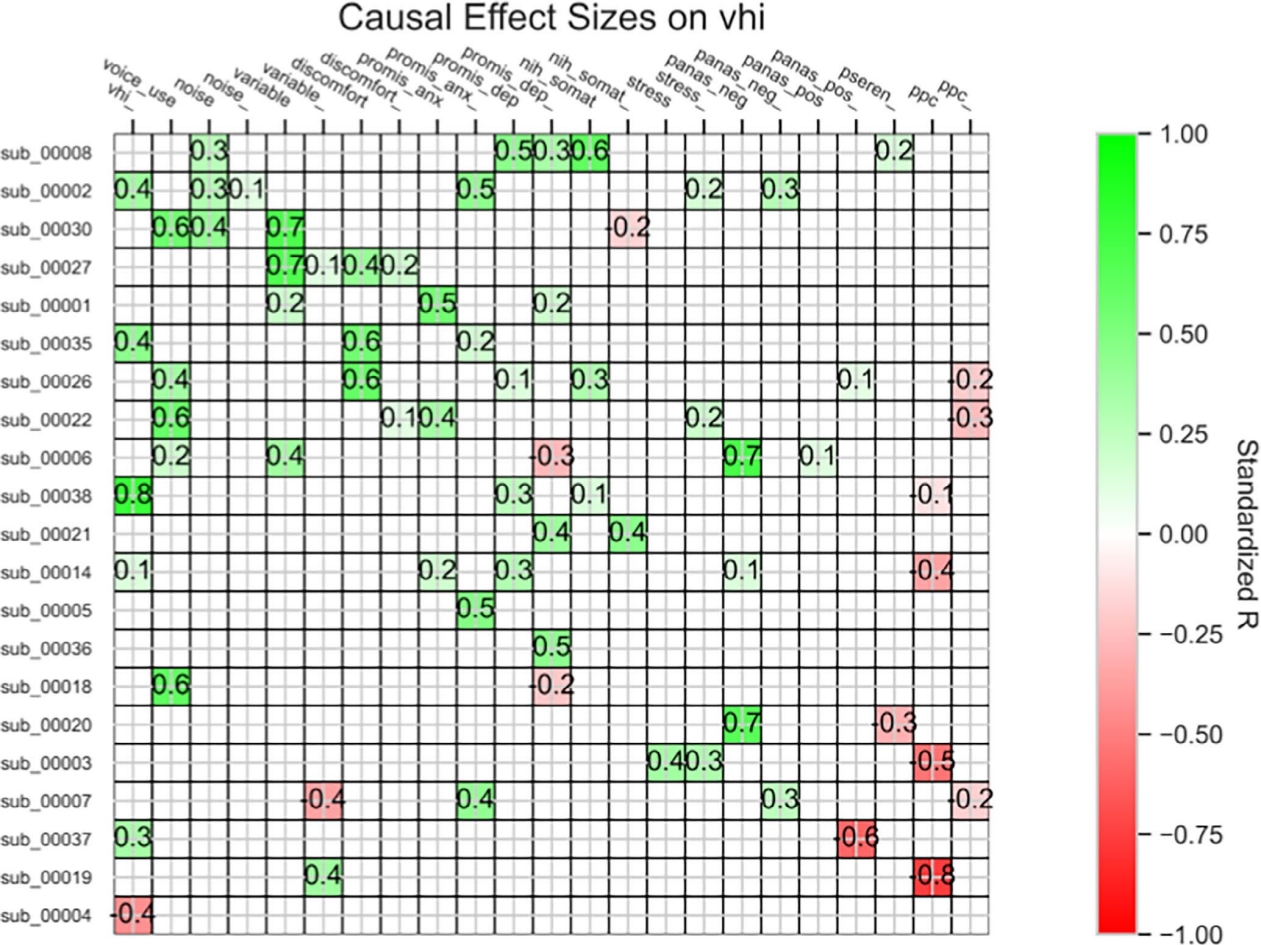
Causal effect sizes on VHI shown for all individual causal models and variables. The color saturation of the cell indicates the strength of the effect, and the color indicates the direction. The number in each box indicates the r. Some individuals had multiple causal factors for voice handicap, while others had very few, and there was a wide range of effect sizes across individuals. nih_somat = somatic arousal, noise = background noise, panas_neg = negative affect, panas_pos = positive affect, ppc = perceived control., promis_anx = anxiety, promis_dep = depression, pseren = serenity, variable = voice variability, vhi = voice handicap. The subscript “_” denotes a prior value of that variable. [Color figure can be viewed in the online issue, which is available at www.laryngoscope.com]

A heatmap grid summary of these causal pathways within and across individuals (Figure 3) offers a visual representation of the heterogeneity across individuals regarding specific factors that were causal for VHI and the magnitude of those effect sizes. For example, in subject 19 (shown in Figure 2 panel A as well as Figure 3 row sub_00019): perceived control (i.e., ppc) strongly decreased voice handicap (vhi) with a highly negative r, shown with strong negative shading in that cell; and prior voice variability (variable_) moderately increased voice handicap (vhi) with a moderately positive r, shown with medium positive shading in that cell. In subject 3 (Figure 2 panel B and Figure 3 row sub_00003): perceived control (ppc) strongly decreased voice handicap (vhi) with a highly negative r, as shown by strong negative shading in that cell; and stress and prior stress (stress_) moderately increased voice handicap (vhi), with moderately positive r's, shown with medium positive shading in those cells. Similar interpretations can be conducted for subjects 38 (Figure 2 panel C and Figure 3 row sub_00038) and 6 (Figure 2 panel D and Figure 3 row sub_00006).

## 4 Discussion

Despite prior literature showing important cross‐sectional associations [5, 7, 8], neither perceived control nor distress predicted subsequent voice handicap in traditional group‐level time series EMA analysis. However, when perceived control and voice handicap were analyzed comparing daytime to evening, perceived control was predictive of subsequent voice handicap. In both analyses, serenity was weakly predictive of subsequent voice handicap. Additional insights were derived from causal modeling. Group‐level causal modeling identified anxiety, but not perceived control or serenity, as causal for voice handicap. Most intriguingly, individual causal analyses identified significant heterogeneity across participants. At the individual level, the algorithm modeled perceived control as a strongly causal factor for voice handicap in some participants. Other factors variably causal for voice handicap in individual‐level models were negative affect, serenity, anxiety, somatic arousal, and stress.

These findings suggest that group‐level analyses of heterogeneous individual‐level experiences, while broadly informative, may produce a different understanding of the relationships between variables. For example, perceived control did not predict subsequent voice handicap in group‐level timepoint‐to‐timepoint analysis; did predict voice handicap in group‐level day‐evening analysis; and was strongly causal for voice handicap in some individuals' models but not in others. This is concordant with a smaller prior EMA study in which multiple phenotypes were identified for the association of the two variables, including (a) no significant variation in either variable, (b) changes in perceived control preceding changes in voice handicap scores, (c) changes in voice handicap scores preceding changes in perceived control, and (d) opposing changes in both variables, such that one increased when the other decreased [10]. These types of heterogeneous findings have shifted thinking in other fields such as mood/alcohol use disorders and eating disorders [14, 15], and may be valuable for approaches to the treatment of voice disorders as well.

Individual heterogeneity may contribute to the variable effects of various interventions [14]. Understanding each individual's causal model may allow improved tailoring of patients' voice care plans. For example, a patient for whom stress is strongly causal for voice handicap may benefit from an intervention focused on reducing stress, whereas someone for whom anxiety is causal might benefit from strategies to reduce anxiety. Future studies could examine whether specific targeting of factors identified as important in individual causal models leads to better outcomes. If successful, clinics could offer early EMA assessments to identify individually relevant intervention targets, leading to personalized treatment components that could be pursued in tandem with speech therapy or other treatments.

Limitations of the study include the modest sample size and sampling time window; given the observed psychological heterogeneity across participants, more participants and timepoints would likely offer more comprehensive insights into potentially important factors in voice care. Limited sociodemographic heterogeneity also reduced generalizability. Other limitations are inherent to the methodology, including the need to use abbreviated scales to avoid response fatigue and the potential for influencing responses by raising awareness of certain factors through repeated assessment. Future studies on this topic could examine whether and how voice therapy and/or severe baseline VHI‐10 scores may influence causal mechanisms. Additionally, this methodology could also be applied to examine the roles of additional psychological factors in voice and other otolaryngologic disorders (e.g., tinnitus, facial pain).

## 5 Conclusions

In group‐level time‐series analyses, predictors of voice handicap varied depending on the examined models. Group‐level causal modeling identified anxiety as causal for voice handicap. Individual‐level analyses identified heterogeneous causal factors for voice handicap, most notably perceived control and negative affect. Group‐level analyses of EMA data may obscure important heterogeneity that can be identified using individual‐level analyses. Causal modeling using intensive longitudinal datasets offers a potential future avenue for designing individualized treatment approaches for voice disorders.

## Conflicts of Interest

The authors declare no conflicts of interest.
